# Optimizing PSMA scintigraphy for resource limited settings – a retrospective comparative study

**DOI:** 10.1186/s40644-024-00693-9

**Published:** 2024-04-01

**Authors:** Olumayowa U. Kolade, Anita Brink, Akinwale O. Ayeni, Stuart More, Jennifer Holness

**Affiliations:** 1 Division of Nuclear Medicine, Department of Radiation Medicine, University of Cape Town, Cape Town, South Africa; 2 Department of Nuclear Medicine, University College Hospital, Ibadan, Oyo State Nigeria; 3 Division of Human Health, Department of Nuclear Sciences and Applications, International Atomic Energy Agency (IAEA), Vienna, Austria; 4 Department of Nuclear Medicine, Klerksdorp/Tshepong Hospital Complex, Klerksdorp, South Africa; 5 Division of Nuclear Medicine, Department of Radiation Sciences, University of the Witwatersrand, Johannesburg, South Africa; 6 Division of Nuclear Medicine, Department of Medical Imaging and Clinical Oncology, Stellenbosch University, Cape Town, South Africa

**Keywords:** ^99m^Tc-PSMA scintigraphy, Resource limitation, Prostate Cancer, Planar, SPECT, SPECT/CT, Developing world

## Abstract

**Background:**

PSMA PET/CT is the most sensitive molecular imaging modality for prostate cancer (PCa), yet much of the developing world has little or no access to PET/CT. [^99m^Tc]Tc-PSMA scintigraphy (PS) is a cheaper and more accessible gamma camera-based alternative. However, many resource-constrained departments have only a single camera without tomographic or hybrid imaging functionality, and camera time is frequently in high demand. Simplifying imaging protocols by limiting the field of view (FOV) and omitting SPECT/CT or even SPECT may provide a partial solution. The aim was thus to determine the adequacy of PS planar-only and/or SPECT-only imaging protocols with a limited FOV.

**Methods:**

The scans of 95 patients with histologically proven PCa who underwent PS with full-body planar and multi-FOV SPECT/CT were reviewed. The detection rates for uptake in the prostate gland/bed and in metastases were compared on planar, SPECT, and SPECT/CT. The agreement between modalities was calculated for the detection of metastases and for staging. The impact of imaging a limited FOV was determined.

**Results:**

Pathological prostatic uptake was seen in all cases on SPECT/CT (excluding two post-prostatectomy patients), 90.3% of cases on SPECT, and 15.1% on planar images (*p* < 0.001). Eleven (11.7%) patients had seminal vesicle involvement on SPECT/CT, which was undetectable/indistinguishable on planar images and SPECT. The agreement between modalities was moderate to good (κ = 0.41 to 0.61) for the detection of nodal metastases, with detection rates that did not differ significantly (SPECT/CT = 11.6%, SPECT = 8.4%, planar = 5.3%). Detection rates for bone metastases were 14.7% (SPECT/CT) and 11.6% (SPECT and planar). Agreement between modalities for the detection of bone metastases was good (κ = 0.73 to 0.77). Three (3.1%) patients had visceral metastases on SPECT/CT, two of which were detected on SPECT and planar. There was good agreement between modalities for the TNM staging of patients (κ = 0.70 to 0.88). No metastatic lesions were missed on the limited FOV images.

**Conclusion:**

When PS scintigraphy is performed, SPECT/CT is recommended. However, the lack of SPECT/CT capabilities should not preclude the use of PS in the presence of limited resources, as both planar and SPECT imaging are adequate and will correctly stage most PCa patients. Furthermore, time-based optimisations are achievable by limiting the FOV to exclude the distal lower limbs.

## Background

Prostate cancer is the second most-frequently diagnosed male malignancy, and represents the fifth foremost cause of cancer associated mortality in men, globally [1, 2]. Developing countries, despite having three-fold-lower incidences compared to high-income countries, have a paradoxically outsized prostate cancer (PCa) mortality burden. Currently, the highest mortality rates of PCa globally are in Sub-Saharan Africa, Micronesia and the Caribbean, all resource-constrained settings [2]. The comparatively favourable PCa outcomes of high-income countries are linked to improved diagnostics and therapeutics [3]. Solutions aimed at optimising diagnosis and treatment for the less-resourced world will therefore have significant impact on bridging the disparity in PCa outcomes.

Bone scintigraphy has been the mainstay nuclear medicine imaging modality to detect bone metastases in PCa for several decades [4–7]. Bone scintigraphy is sensitive, however has some drawbacks including lower specificity and the inability to detect visceral and nodal metastases [8].

In recent times, new molecular tracers have been developed that target the transmembrane glycoprotein Prostate Specific Membrane Antigen (PSMA), which is over-expressed in PCa cells and metastases [9]. This is done using positron emitters like ^68^Ga and ^18^F in positron emission tomography/computed tomography (PET/CT) imaging - PSMA PET. PSMA PET is well established as being superior to conventional imaging for staging, recurrence detection, patient-selection and planning for peptide receptor radioligand therapy (PRLT), and post-therapy response assessment in PCa [5, 10–15]. PSMA PET is however out of reach for most of the developing world, as 92–95% of low-to-lower-middle-income countries do not have a PET/CT unit [16, 17]. Furthermore, the cost of PET/CT is frequently prohibitive. For example, PSMA PET studies, where available across Africa, currently cost between 800 to 1000 United States Dollars (USD) per patient [18, 19].

[^99m^Tc]Tc-PSMA scintigraphy (PS) utilises the same molecular target (PSMA), but with the more affordable and widely available radioisotope ^99m^Tc. In contrast to bone scintigraphy, PS can detect extra-osseus metastases in addition to bone metastases [20]. [^99m^Tc]Tc-PSMA is imaged with a gamma camera, and planar as well as Single Photon Emission Computed Tomography (SPECT) images may be recorded. Hybrid imaging with Single Photon Emission Computed Tomography with Computed Tomography (SPECT/CT) is also possible with a SPECT/CT gamma camera. Gamma cameras are cheaper and more readily accessible in the developing world compared to PET/CT. The cost of a SPECT/CT camera is higher than that of a standalone gamma camera but less than a PET/CT camera. Although [^99m^Tc]Tc-PSMA is also costly, approximately USD 300 per patient locally, PS enables the clinical utilisation of PSMA-targeted imaging without the enormous capital demands of PET/CT camera acquisition, installation, and maintenance, as well as the high costs of PET radiopharmaceuticals. PS therefore represents a potential alternative and possible solution for improving PCa imaging in resource limited departments and centres with no access to PET/CT.

Early work comparing PS SPECT to PSMA PET (without CT) found PET to be significantly more sensitive than SPECT [21]. However, studies directly comparing PS SPECT/CT to PSMA PET/CT found no significant difference in the detection rates of skeletal metastases, thus concluding PS SPECT/CT to be sufficiently comparable to PSMA PET/CT in this regard [20, 22]. The utility of PS has been evaluated almost exclusively using multi field-of-view (FOV) SPECT/CT and there is a paucity of literature assessing the adequacy of planar imaging or SPECT alone in PS [20, 22–29]. One pilot study of 18 patients with PCa, which primarily compared PS with PSMA PET, also analysed the comparative lesion detection rates of PS on planar imaging versus SPECT and SPECT/CT. They found superior lesion detection with SPECT and SPECT/CT compared to planar imaging. They also reported poor planar-to-SPECT/CT agreement, fair planar-to-SPECT agreement and good SPECT-to-SPECT/CT agreement [30].

The practical reality, however, is that many nuclear medicine centres across the developing world do not have access to hybrid imaging, with only planar gamma cameras or at best those with SPECT capabilities [31, 32]. These resource limitations are compounded by a higher population per camera compared to the developed world. As an example, Nigeria (Africa’s most populous nation of approximately 200 million people, 50% of whom live on less than two dollars per day) has only three fully functioning nuclear medicine centres with four gamma cameras between them, and a dearth of specialised personnel. Most patients need to travel long distances to access pooled and infrequently provided radionuclide studies, thus negatively impacting the delivery of nuclear medicine services [31–35].

Therefore, strategies for optimising camera utilization are essential. A potential partial solution is to shorten imaging protocols, by objectively defining the acquisition steps that are essential and those that may be done away with, without significantly impacting study interpretation and patient management. Thus, the aim of this study was to determine the adequacy of simplified/truncated imaging protocols for [^99m^Tc]Tc-PSMA scintigraphy, by comparing the detection rates for PCa on planar imaging, multi-FOV SPECT and multi-FOV SPECT/CT. This study was designed to test the hypothesis that planar, and SPECT imaging will adequately and correctly stage most patients compared to SPECT/CT.

## Methods and materials

### Study Design & Setting

A retrospective review of consecutive patients with histologically proven PCa who had [^99m^Tc]Tc-PSMA scans performed at the Nuclear Medicine Division of Groote Schuur Hospital, University of Cape Town, South Africa between January 2018 and December 2021.

The inclusion criteria were patients older than 18 years with histologically proven PCa who were imaged with both whole-body PS planar and three-volume SPECT/CT (from vertex to mid-thighs). The studies were requested either for staging of high-risk or high-tier intermediate risk disease, as well as evaluation prior to potential [^177^Lu]Lu-PSMA therapy.

Patients with a history of a secondary malignancy at the time of PS, incomplete scanning protocols (e.g. due to camera problems), non-contiguous SPECT volumes, missing images (e.g. due to problems with archiving of old studies), and studies identified with technical problems were excluded. In cases where patients underwent PS on two or more occasions, only the first of the scans was included.

### Ethical considerations

Ethical approval was obtained from the Human Research Ethics Committee of the University of Cape Town (HREC REF: 724/2021) and hospital approval was obtained from the management of Groote Schuur Hospital.

### Radiopharmaceutical

The [^99m^Tc]Tc-PSMA was prepared by external laboratories, supplied as single patient doses with quality control certificates and administered within stated stable period (4 hours); in-house quality control was not repeated. The radiopharmaceutical was either [^99m^Tc]Tc-PSMA T4 or [^99m^Tc]Tc-PSMA I&S. The activity ordered was 750 MBq per patient. Mean injected activity (mean ± SD) was 750 ± 50 MBq.

### Image acquisition protocol

Images were acquired four hours after radiotracer administration on a Siemens e.Cam Signature and Siemens Symbia T6 SPECT-CT gamma cameras (Siemens Medical Solutions SW, Erlangen), both with low-energy high-resolution collimators.

Planar images were acquired as whole-body sweep images at a speed of 14 cm per minute, on a 1024 × 256 matrix, with a zoom of 1.0 with auto-contouring, using anterior and posterior detectors.

Subsequently, SPECT/CT images were acquired on the dedicated Siemens Symbia T6 SPECT-CT (Siemens Medical Solutions SW, Erlangen) in three volumes/bed-positions (multi FOV) covering vertex to mid-thighs (380 mm per bed position) on a 128 × 128 matrix, at a zoom of 1.00, on a non-circular orbit, in step-and-shoot mode (3^o^ angles) for 20 seconds per view. A low-dose non-contrast enhanced CT scan acquired for attenuation correction and anatomical localisation; CT parameters utilised 30 mAs, 120 kV, at a pitch of 1.5 and a slice thickness of 3 mm.

### Image processing protocol

Imaging studies were reconstructed and processed on dedicated Hermes physicians’ workstations using Hermes Gold Lx Browser (version 2.15, 2022; Hermes Medical Systems, Sweden) to ensure standardised reconstruction parameters for SPECT and SPECT/CT. SPECT images were reconstructed utilising ordered subset expectation maximisation (OSEM) with five iterations, 16 subsets, and a 0.9 cm Gaussian full width half maximum (FWHM) postfilter. Resolution recovery and scatter correction were also applied. SPECT images for review under the SPECT-only protocol were processed without attenuation correction. SPECT images for SPECT/CT review were reconstructed utilising CT-attenuation correction, and images were viewed utilising the ‘B31s’ Siemens CT kernel.

### Imaging analysis

A visual analysis of the images was performed by two nuclear medicine physicians (nine- and four-years’ experience respectively), and one nuclear medicine trainee (final year). They were blinded to the patients’ clinical history, radiological and biochemistry results. Interpretation data was entered into a standardised data-capture sheet at each point.

Interpretation was approached in a three-stage, sequential manner: First, all whole-body planar images were interpreted, followed by all multi-FOV SPECT-only images, and finally the multi-FOV SPECT/CT-only images. There were at least two-week intervals between subsequent stages, and images were reviewed in random order to eliminate recall bias. The observers specifically assessed the prostate gland or bed, seminal vesicles, lymph nodes, bones, and viscera. Each site was assessed as being positive, negative, or equivocal for [^99m^Tc]Tc-PSMA uptake. Lymph node uptake was further identified as being present within the loco-regional drainage basin of the prostate or in distant sites; the number of positive lymph nodes was not counted in each patient. Identified bone lesions were counted and designated oligometastatic if five or fewer sites were involved, or designated multiple if more than five [36, 37]. Visceral metastases were identified and localised.

Images were initially interpreted by each reviewer individually, and where incongruities existed, by consensus across all three viewers for each imaging modality. Consensus reading was also performed in random order to eliminate recall bias. Images on which no agreement was reached were indicated as ‘equivocal’ in the data set.

Consensus reports of planar-only, SPECT-only, and SPECT/CT-only images were then compared for each patient.

### Data analysis

Using patient-based analyses, the detection rates of prostatic uptake, seminal vesicle involvement, lymph node metastases, bone metastases and visceral metastases were calculated and compared for SPECT/CT, SPECT and planar. SPECT/CT served as the reference method for distinguishing between true positive and false positive cases on SPECT and planar. The scan-based American Joint Committee on Cancer (AJCC) / Union for International Cancer Control (UICC) 8th edition TNM stage of each patient was determined and compared for planar, SPECT and SPECT/CT [38, 39]. The number of cases that were incorrectly upstaged or downstaged by planar and SPECT were determined by comparison with SPECT/CT. The agreement (Cohen’s Kappa) between planar, SPECT and SPECT/CT was determined for the detection of lymph node and bone metastases, and for TNM staging [40]. The number of lesions missed by analysing a limited FOV (vertex-to-mid-thigh) was determined. Statistical analysis was performed using MedCalc® Statistical Software version 22.009 (MedCalc Software Ltd., Ostend, Belgium; https://www.medcalc.org; 2023).

## Results

During the period under review, 306 [^99m^Tc]Tc-PSMA studies were performed in 303 patients. Following the exclusion of scans without both planar and tomographic acquisitions (208), incomplete data sets (1), and repeat studies (2), 95 patients were included.

The median age of the cohort was 66 years, with ages ranging from 47 to 80 years. Ninety-one patients had PS for staging purposes, and the remaining four underwent PS as part of the workup for [^177^Lu]Lu-PSMA therapy.

The dose of [^99m^Tc]Tc-PSMA administered to the patients, measured shortly before administration, ranged between 700 and 800 MBq (750 ± 50 MBq).

### Prostate gland and seminal vesicle involvement

Two patients had undergone prostatectomy prior to PS (one radical prostatectomy; one simple prostatectomy). On SPECT/CT, there was abnormal uptake in the prostate of all other 93 patients.

**Fig. 1:**
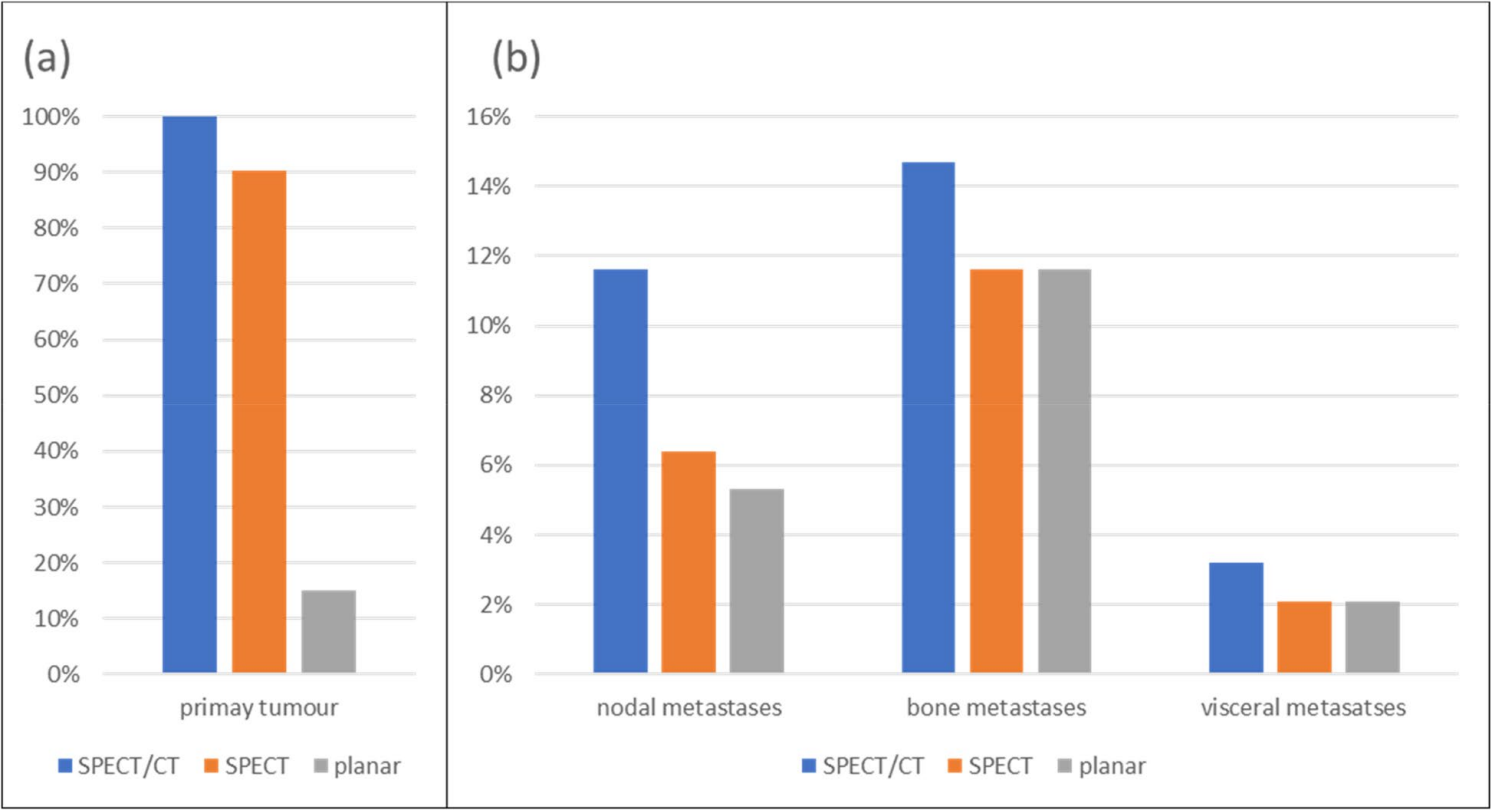
A comparison of the detection rates on SPECT/CT, SPECT, and planar imaging for (**a**) the primary tumour and (**b**) lymph node metastases, bone metastases and visceral metastases

On planar imaging, pathological prostatic uptake was detected in 14/93 cases (15.1%) (Fig. 1). The observers were unable to assess for abnormal prostatic PSMA uptake in the remaining 79 (84.9%) patients.

SPECT correctly detected 84/93 (90.3%) cases with abnormal prostatic uptake. There was one false negative case on SPECT, and eight (8.6%) SPECT cases were equivocal for prostate involvement. SPECT/CT detected significantly more cases of prostatic involvement than both SPECT (*p* = 0.002) and planar images (*p* < 0.001). SPECT also detected significantly more cases than planar (p < 0.001) .

**Fig. 2:**
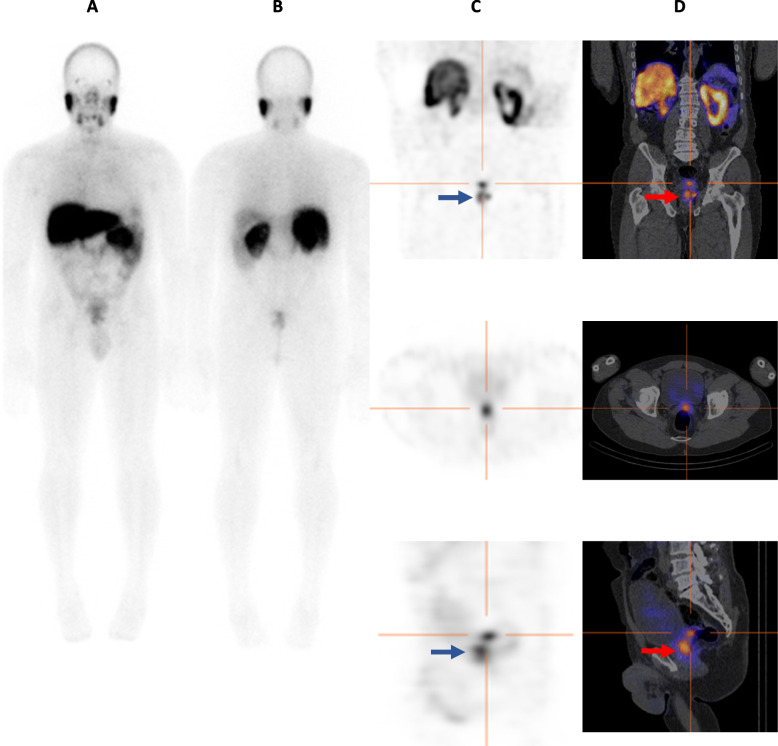
On planar images (**A**, **B**) it is difficult to distinguish activity in the pelvis as being prostatic uptake or urinary bladder activity. On SPECT images (**C**) uptake is confidently attributed to the prostate (tip of blue arrows on coronal and sagittal views), with an adjacent focus (**C**; crosshairs on coronal, trans-axial, and sagittal views) that observers queried as being in an adjacent lymph node or seminal vesicle. On SPECT/CT (**D**) uptake is localized to the prostate gland (tip of red arrows) and the left seminal vesicle (crosshairs)

On SPECT/CT, 11/94 cases (11.7%) were positive for seminal vesicle involvement and 83/94 cases (88.3%) were negative. Seminal vesicle involvement was impossible to determine on planar imaging in all patients. SPECT alone was unable to assess seminal vesicles in 93/94 (98.9%) of the patients. Figure 2 is an example of a case in which there was disagreement between observers on uptake being in seminal vesicle or in an adjacent lymph node.

### Nodal involvement

**Fig. 3:**
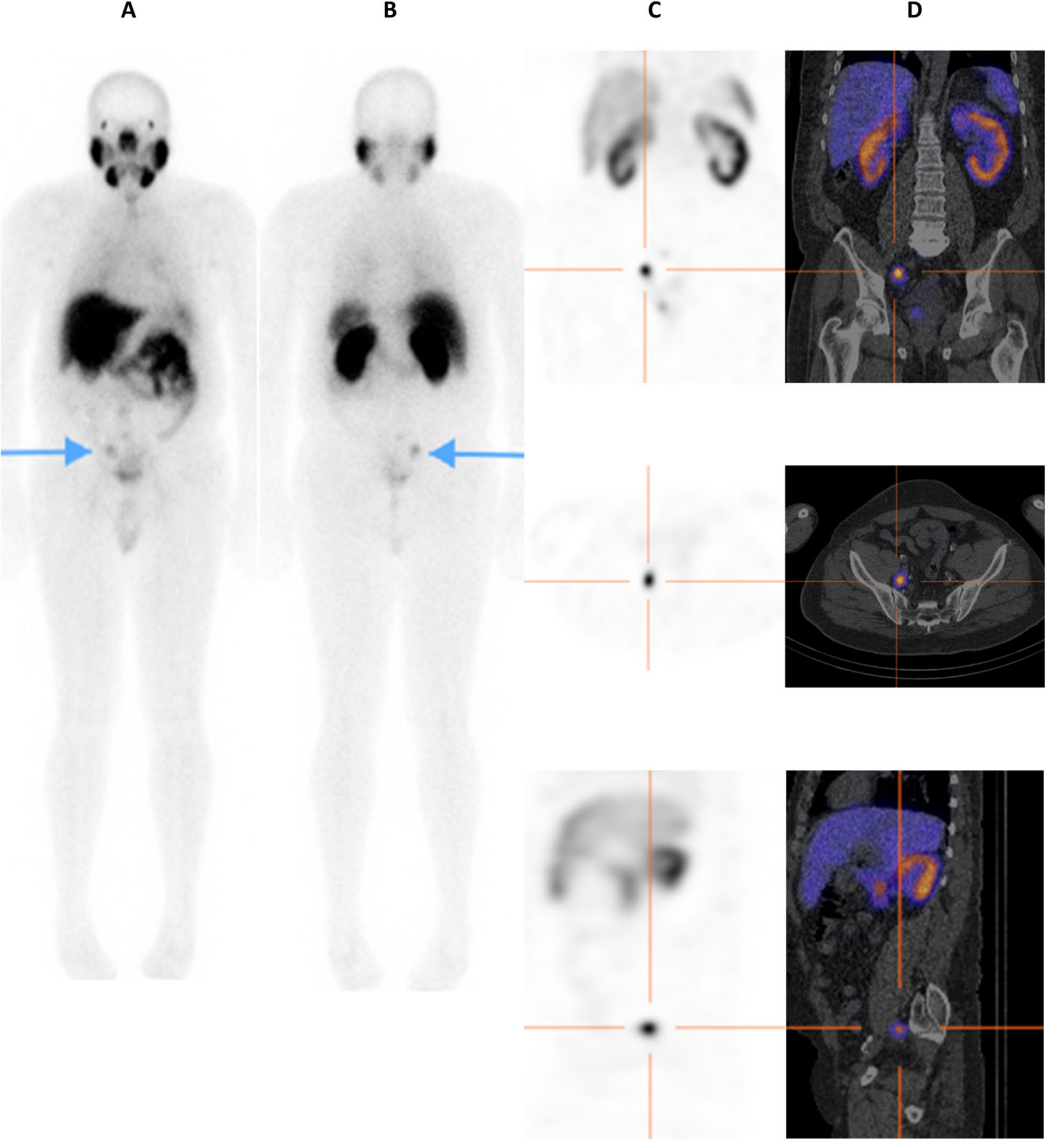
An example of agreement on planar images (**A** & **B**- tip of arrows on anterior and posterior projections), SPECT (**C**; crosshairs), and SPECT/CT (**D**; crosshairs) in detecting a loco-regional (right internal iliac) lymph node

The assessment of lymph node involvement was consistent on planar, SPECT, and SPECT/CT in 71/95 (74.7%) patients - 65 were negative, five positive, and one equivocal. Moderate agreement was found between planar and SPECT (κ = 0.41; 95% CI 0.20–0.61), fair agreement between planar and SPECT/CT (κ = 0.40; 95% CI 0.20–0.59) and good agreement between SPECT and SPECT/CT (κ = 0.61; 95% CI 0.41–0.80). Figure 3 is an example of a case in which assessment of lymph node metastases was consistent across all three modalities.

On SPECT/CT PSMA-positive nodal disease was detected in 11/95 (11.6%) patients, 83 (87.4%) were negative for nodal disease, and one case was classified as equivocal (Table 1, Fig. 1). The equivocal case had abnormal uptake of moderate intensity in a distant node without loco-regional nodal disease. The distribution was not typical for metastatic PCa, and it was agreed lymph node biopsy would be needed to confirm/exclude metastatic disease.

**Table 1:** Lymph node metastases detection rates on planar, SPECT, and SPECT/CT (*n* = 95)

	SPECT/CT^b^	Planar	SPECT	***p***-value
Positive cases^a^	11 (11.6%)	5 (5.3%)	8 (8.4%)	0.12 to 0.46
Negative cases	83 (87.4%)	71 (74.7%)	75 (78.9%)	0.03^d^ to 0.49
False positive	N/A	1 (1.1%)^b^	1 (1.1%)^b^	N/A
Equivocal cases	1 (1.1%)^c^	15 (15.9%)	10 (10.5%)	< 0.001^e^ to 0.27

^a^ Positive cases = locoregional + distant nodal metastases

^b^ SPECT/CT assessment used as reference method

^c^ Abnormal uptake was present in a lymph node, but it was equivocal for metastatic disease

^d^ Significant difference found for SPECT/CT vs. planar

^e^ Significant difference found for SPECT/CT vs. planar and SPECT/CT vs. SPECT

Planar imaging detected nodal metastases in 5/95 (5.3%) patients and identified 71/95 (74.7%) nodal negative cases. Fifteen (15.8%) cases were deemed equivocal for nodal involvement. There was one false positive case (Table 1).

**Fig. 4:**
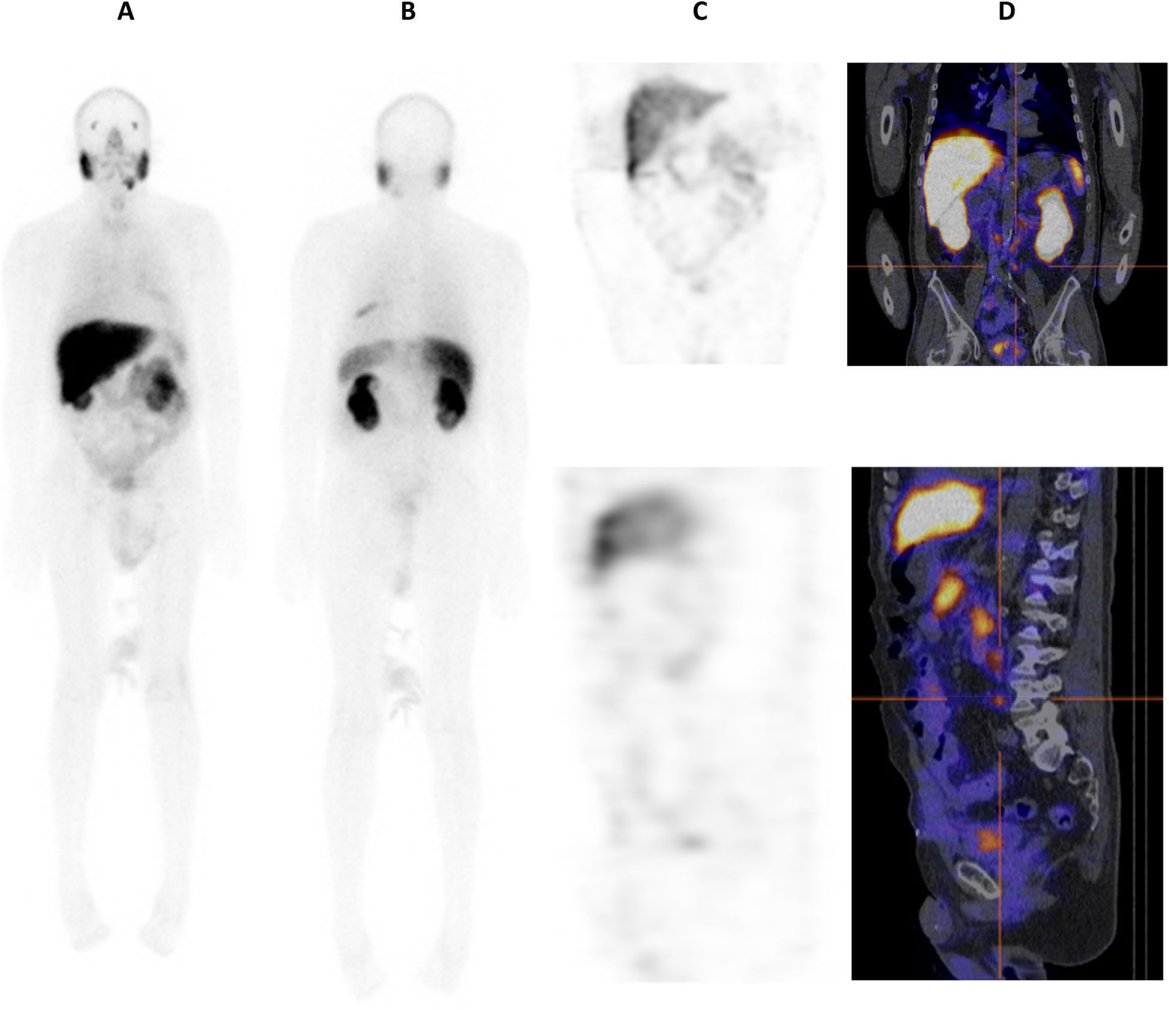
An example of uptake detected in a para-aortic lymph node on SPECT/CT (cross hairs on **D**) but missed on planar (**A**,**B**), and SPECT images (**C**). Linear right rib uptake noted on the posterior planar projection (**B**) was also concordant on SPECT and SPECT/CT (outside frame of zoomed image)

SPECT images detected 8/95 (8.4%) patients with nodal metastases, and 75/95 (78.9%) cases were negative. Ten (10.5%) cases were equivocal. There was one false positive on SPECT (Table 1). The false positive case on both planar and SPECT was confirmed on SPECT/CT to be focal skeletal uptake in the ilium. Figure 4 is an example of a patient with a metastatic para-aortic lymph node that was not detected on planar or SPECT imaging,

SPECT/CT did not detect significantly more patients with nodal metastases than either planar (*p* = 0.12) or SPECT (*p* = 0.46). Similarly, the detection rate for nodal metastases was not significantly different on planar and SPECT (*p* = 0.40). The proportion of equivocal cases on SPECT and planar did not differ significantly (*p* = 0.27) but there were significantly more equivocal cases on planar (p = < 0.001) than SPECT/CT, and SPECT than SPECT/CT (*p* = 0.006).

Of the 11 cases with positive nodal disease on SPECT/CT, seven had disease confined to loco-regional nodes, and four had distant nodal metastases. In all 5 of the patients identified as having nodal metastases on planar, and in all 8 on SPECT, the distinction of loco-regional involvement vs. distant nodal metastases was correctly made.

### Skeletal involvement

All patients were assessed for bone metastases. In most patients (86/95; 90.5%), skeletal findings on planar, SPECT, and SPECT/CT were consistent. There was good agreement between planar and SPECT (κ = 0.77; 95% CI 0.62–0.92), between planar and SPECT/CT (κ = 0.76; 95% CI 0.59–0.94) and between SPECT and SPECT/CT (κ = 0.73; 95% CI 0.54–0.91).

SPECT/CT detected 14/95 (14.7%) patients with PSMA-positive skeletal metastases (Fig. 1), of whom six had oligometastatic disease, and eight had uptake in more than five skeletal sites. The remaining 81 cases (85.3%) were negative for bone metastases. There were no equivocal cases on SPECT/CT.

**Fig. 5:**
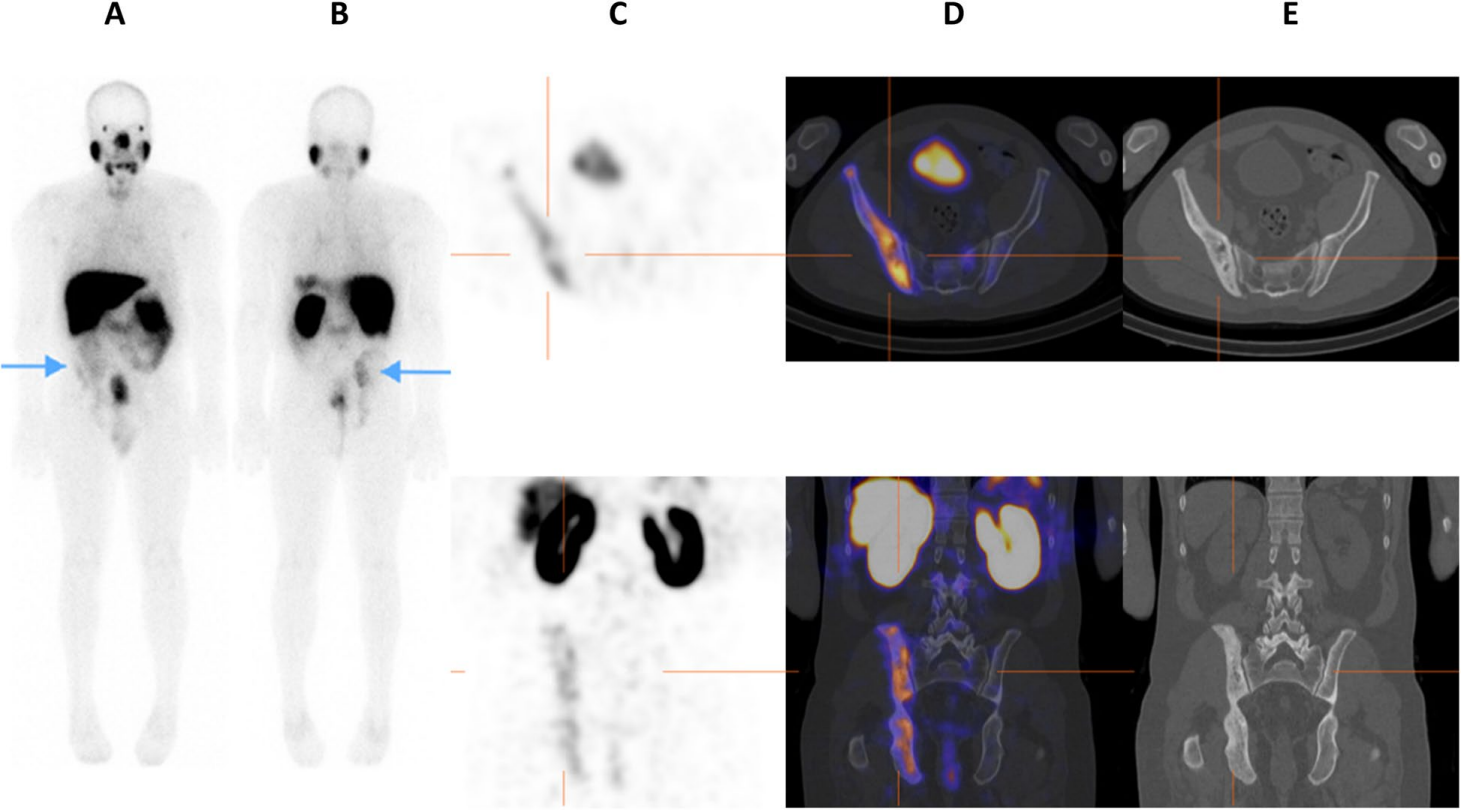
False positive oligometastatic skeletal uptake in the right hemi-pelvis on planar images (**A** & **B**- tip of arrows on anterior and posterior projections), and SPECT images (**C** - crosshairs on coronal and trans-axial views). Characterization of uptake on SPECT/CT (**D**) and CT only views (**E**) - was typical of Paget’s disease with associated cortical thickening, bony expansion, and coarsened trabeculae

On planar imaging, 11/95 cases (11.6%) with bone metastases were detected, and 78/95 (82.1%) of cases were negative for bone metastases (Table 2). There was one false positive case and three that were equivocal. The false positive case was confirmed on SPECT/CT to be Paget’s Disease in the ilium (Fig. 5).

**Table 2:** Bone metastases detection rates on planar, SPECT, and SPECT/CT (*n* = 95)

	SPECT/CT	Planar	SPECT	***p***-value
Positive cases^a^	14 (14.7%)	11 (11.6%)	11 (11.6%)	0.53 to 1.0
Negative cases	81 (85.3%)	78 (82.1%)	77 (81.1%)	0.44 to 0.86
False positive	N/A	1 (1.1%)^b^	3 (3.2%)^b^	0.32^c^
Equivocal cases	0	3 (3.2%)	2 (2.1%)	0.08–0.64

^a^True positive cases = oligometastatic cases + cases with > 5 skeletal lesions

^b^SPECT/CT assessment used as reference method

^c^*P*-value calculated for planar vs. SPECT

SPECT also detected 11/95 (11.6%) positive cases and identified 77/95 (81.1%) that were negative. There were three false positive and two equivocal cases on SPECT (Table 2). The three false positive cases were lesions related to Paget’s Disease (2), and oesophageal activity falsely designated as vertebral uptake (1).

The detection rates for bone metastases on SPECT/CT, SPECT and planar were not significantly different (*p* = 0.53 to 1.0). The proportion of negative cases did not differ significantly between planar, SPECT and SPECT/CT (*p* = 0.44 to 0.86). There was also no significant difference in the proportion of equivocal cases between SPECT/CT, SPECT and planar (*p* = 0.08 to 0.64) (Table 2).

On planar imaging the distinction between oligometastatic disease and patients with > 5 lesions was correctly made in all 11 cases. On SPECT one case with oligometastatic disease was incorrectly classified as having multiple metastases.

### Visceral involvement

On SPECT/CT three patients (3.2%) were identified with visceral (lung) metastases. Planar imaging and SPECT both identified two of the three patients (Fig. 1). The third case was equivocal on planar imaging but falsely negative on SPECT.

**Fig. 6:**
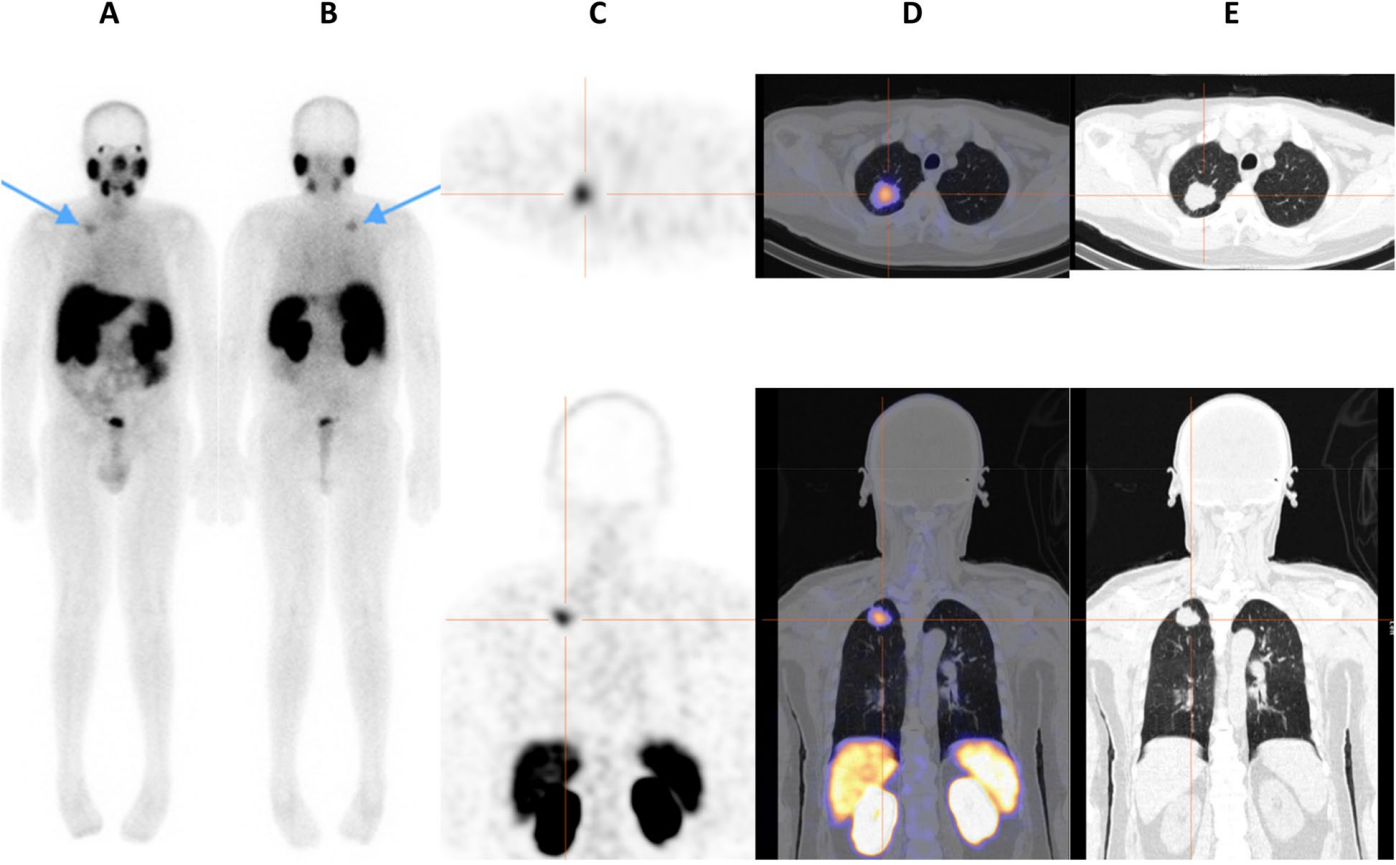
PSMA avid lesion was incorrectly reported as a rib metastasis on planar imaging (**A** & **B**- tip of arrows on anterior and posterior projections) and as a lung metastasis on SPECT images (**C** - crosshairs on trans-axial and coronal views). Right lung lesion on SPECT/CT (**D**) with corresponding CT findings (**E**) were suspicious for a second primary malignancy. Biopsy confirmed a primary lung adenocarcinoma

A lung lesion was seen on SPECT/CT in an additional patient. Corresponding CT findings in this case raised suspicion for a second primary malignancy. Subsequent biopsy confirmed a primary lung adenocarcinoma. This lesion was incorrectly reported as a rib metastasis on planar imaging and as a lung metastasis on SPECT (Fig. 6).

### Staging

SPECT/CT classified 16 (16.8%) patients as having stage IVB disease, 6 (6.3%) with stage IVA disease, 4 (4.2%) with stage IIIB/C disease, and 69 (72.6%) with ≤ stage IIIA (i.e. (T1 or T2, N0, M0) disease (Table 3). The agreement between all modalities for staging of patients was good: κ = 0.70 for planar vs. SPECT/CT (95% CI 0.54–0.86); 0.74 for SPECT vs. SPECT/CT (95% CI 0.60–0.89); κ = 0.88 for planar vs. SPECT (95% CI 0.76–1.00).

**Table 3:** TNM Staging on planar, SPECT, and SPECT/CT

	SPECT/CT	Planar	SPECT
**Stage** ***≤*** **IIIA**^**a**^	**69 (72.6%)**	**77 (81.1%)**	**73 (76.8%)**
**Stage IIIB/C**	**4 (4.2%)**	**0**	**0**
**Stage IVA**	**6 (6.3%)**	**5 (5.3%)**	**5 (5.3%)**
**Stage IVB**	**16 (16.8%)**	**13 (13.7%)**	**17 (17.9%)**
**Incorrectly staged**^b^	**N/A**^b^	**11 (11.6%)**	**10 (10.5%)**
Downstaged:	N/A^b^	10 (10.5%)	7 (7.4%)
IIIB/C → ≤ IIIA		4	4
IVA → ≤ IIIA		2	1
IVB → ≤ IIIA		3	1
IVB → IVA		1	1
Upstaged:	N/A	1 (1.1%)	3 (3.2%)
≤IIIA → IVB		1	2
IVA → IVB		0	1

^**a**^ Prostate scintigraphy cannot distinguish between stages IIIA and lower

^b^ SPECT/CT assessment used as reference method to determine upstaged and downstaged cases on planar and SPECT

On planar imaging and SPECT, the staging was the same as on SPECT/CT in 84/95 (88.4%) and 85/95 (89.5%) of patients respectively. Using SPECT/CT as the reference method, planar incorrectly downstaged 10 (10.5%) patients and upstaged 1 (1.1%). SPECT incorrectly downstaged 7 (7.4%) and upstaged 3 (3.2%) (Table 3).

### Lesions outside ‘vertex-to-thigh’ field of view

Based on assessment of the whole-body planar images, of the 95 patients, three had abnormal [^99m^Tc]Tc-PSMA uptake outside a ‘vertex-to-upper-thigh’ field of view. None of these lesions were metastatic. Two patients had degenerative changes in the knee joints, and one had lesions typical of Paget’s disease.

## Discussion

In this study the detection rates for the primary tumour, seminal vesicle involvement and metastatic disease were compared on 3-volume SPECT/CT, 3-volume SPECT, and whole-body planar imaging in 95 patients. SPECT/CT and SPECT were superior to planar imaging for the detection of uptake in the primary tumour. Only SPECT/CT could detect extension of disease into the seminal vesicles. Although the number of patients with lymph node and bone metastases was higher on SPECT/CT, the proportions detected did not differ significantly between SPECT/CT, SPECT and planar imaging. Three patients had visceral metastases on SPECT/CT, of which both SPECT and planar imaging detected two cases. The agreement between the three imaging modalities ranged from fair to good for the detection of nodal metastases. However, there was good agreement between modalities for the detection of bone metastases and for the TNM staging of patients.

Although PSMA PET/CT is the most sensitive nuclear medicine imaging modality for PCa, PS is arguably comparable and better suited for the developing world [20, 22, 41]. PS, however, has mostly been utilised within the context of hybrid imaging (with SPECT/CT) which is out of reach for most centres in the developing world [20, 22–29, 31, 32]. Therefore, comparative evaluation of planar imaging and SPECT versus SPECT/CT in PS will help optimise the utility of PSMA radioligand imaging in the resource constrained world.

In a pilot study of 18 patients, Vangu et al. compared overall lesion-detection on planar, SPECT and SPECT/CT PS (as a secondary aim within their comparative analysis of PS versus PSMA PET/CT) [30]. They found significantly higher lesion detection with SPECT and SPECT/CT, compared to planar PS; however, they did not specify the nature/site of the lesion identified. They also found moderate agreement between SPECT and SPECT/CT, but significant disagreement between planar and SPECT/CT PS. These findings were comparable to ours for the assessment of prostatic PSMA uptake, wherein we found an inadequacy of planar imaging to detect prostatic disease (15.1% detection rate), but a significantly better detection rate on SPECT (90.3%, *p* < 0.001). The inadequacy of planar imaging to assess prostatic uptake is largely due to the inability to distinguish prostatic uptake from urinary bladder activity superiorly, and bowel activity posteriorly. We note that all patients in our cohort with a prostate in situ had PSMA positivity in the primary tumour. This is in contrast with existing literature, wherein 4.1–10% of cases are found to be PSMA-negative [42, 43]. We did not investigate the reason for this finding but postulate it it may be due the population characteristics of our cohort or the advances stage of disease in the majority of patients. However further investigation with larger prospective studies will be required for substantiation.

In the current study, seminal vesicle involvement was detected in 11.5% of cases on SPECT/CT. As only five patients underwent subsequent radical prostatectomy, numbers were too small to determine the diagnostic accuracy, and no similar published data could be found using PS. However, two retrospective reviews, each of 21 patients who underwent PSMA PET/CT with subsequent histopathological correlation, found reasonable accuracy for detecting seminal vesicle involvement. The sensitivities were 73 and 75% respectively, specificities and positive predictive values were 100% in both studies, and negative predictive values were 77 and 97% respectively [44, 45]. It is expected that the sensitivity of PS SPECT/CT would be lower than that of PET/CT. Of note, in our study, it was not possible to discern seminal vesicle involvement at all on planar imaging or SPECT due to the lack of anatomical landmarks for localization.

The utility of PSMA-radioligand imaging for the T-staging of PCa has been studied almost exclusively within the context of PSMA PET/CT, and available data demonstrates a limited role in this regard due to the superior accuracy of multiparametric magnetic resonance imaging [46, 47]. Thus, considering the lower accuracy of PS, it is unlikely that PS will ever play a significant role in the T-staging of PCa [20, 21]. However, given the good detection rate for PSMA-positive prostatic disease on SPECT PS (90.3%), it may have utility in identifying patients with PSMA-negative disease if no pathological uptake is demonstrated [48]. This is especially applicable in patients with a high pre-test probability of metastatic disease. Additionally, if seminal vesicle involvement *is* detected, management decisions may be impacted as this indicates locally advanced (TNM stage IIIB) disease. Patients with locally advanced disease have been found to have better outcomes with radical radiotherapy rather than radical prostatectomy which is performed for organ-confined disease [5, 49–51].

In our patient cohort, 11/95 (11.6%) had nodal metastases that were detected on SPECT/CT. Seven of these had disease confined to locoregional nodes and four had distant nodal metastases. The number of nodal positive cases detected on SPECT (8) and planar (5) did not differ significantly (*p* = 0.34). Although only one case was equivocal on SPECT/CT, this was not significantly lower than the proportion of equivocal cases on planar (15.9%) or SPECT (10.5%). A retrospective review by Schmidkonz et al. of 93 scans using a different Tc-99 m-based PSMA tracer (^99m^Tc-MIP-1404) found only one case (with two nodes) with histologically confirmed PSMA avid nodal disease, in contrast to ours with 11 cases. Additionally, of the 312 nodes that were histologically sampled in their study and confirmed as negative, none demonstrated uptake on PS, hence were true negatives [52]. The difference in detection rates between the two studies likely primarily reflects differences in the patient populations. Comparability with our findings is further limited by the absence of histological correlation in ours, as well as the absence of inter-modality comparison in their study.

We highlight that in 74.5% of cases the assessment of lymph node involvement was consistent on planar, SPECT and SPECT/CT. While the agreement between planar and SPECT/CT was only fair (κ = 0.40), the agreement between SPECT and SPECT/CT was good (κ = 0.61). Furthermore, there was no upstaging or downstaging from loco-regional to distant nodal disease (N1/N2 to M1 disease) on planar and SPECT, when compared to SPECT/CT. This implies that in our patients with PSMA-positive nodal uptake on planar PS, additional imaging would not have changed clinical management.

Planar, SPECT and SPECT/CT concurred in > 90% of cases for the detection of PSMA-avid skeletal metastases and the agreement between all modalities was good (κ = 0.72 to 0.77). Fourteen (14.7%) patients were found to have bone metastases on SPECT/CT. This was not significantly higher than the detection rates on planar and SPECT that each detected 11/95 (78.6%). A study conducted by Schmidkonz et al. in 2020, primarily aimed to assess interobserver variability in PS, also compared inter-modality agreement between planar and SPECT/CT [53]. They observed better agreement in assessing skeletal lesions compared to the agreement for nodal findings, which is consistent with our findings.

In our cohort, there were three patients (3.2%) with visceral metastases. Detection rates reported in the literature vary. Li et al., in their cohort of 147 patients with PS studies, reported a detection rate of 2% for visceral metastases (pulmonary and hepatic), whilst Sergieva et al. found three of 21 patients (14.3%) with pulmonary, hepatic and adrenal metastases on PS [54, 55]. These were reported on SPECT/CT, however no mention was made on comparative assessments with planar or SPECT studies. In our study, both planar imaging and SPECT correctly detected 2/3 of the cases with visceral metastases. An additional patient had a lung nodule on SPECT/CT. Based on the intensity of the PSMA uptake, it was indistinguishable from metastatic PCa, however radiological features raised suspicion of a primary lung tumour. It was later confirmed histologically to be a primary lung adenocarcinoma. This lesion was reported as a PCa lung metastasis on SPECT and as a bone metastasis (in a rib) on planar imaging.

We did not detect any metastatic lesions outside the vertex-to-upper-thigh field of view on planar imaging. Therefore, we postulate that imaging beyond the level of upper thighs is not essential, especially in a resource limited setting where camera-time is a critical resource. This agrees with the observations of a prospective multicenter study that assessed the clinical relevance of lesions missed by a reduced field of view PET/CT, compared to true whole-body acquisition, and found few missed lesions which had no impact of clinical management changes if captured [56]. This study was however conducted in paediatric lymphoma patients as opposed to our PCa patient population hence direct comparability is limited, given the pathologic and demographic context of our work.

Treatment decisions are based largely on the TNM staging of the patient and therefore accurate staging is of paramount importance. We found good agreement between modalities for the clinical staging of patients (κ = 0.74 to 0.88). Using the SPECT/CT TNM stage as reference, planar imaging correctly staged 84/95 patients (88.4%), and SPECT correctly staged 85/95 (89.5%).

The superiority of SPECT/CT over SPECT and planar imaging is well established in the literature, especially in the context of bone scintigraphy, where it has been extensively studied [57–62]. This is also reflected in the results of the current study where SPECT/CT was clearly superior for the detection of prostatic uptake and seminal vesicle involvement, and it detected a larger number of patients with lymph node, bone and visceral metastases. Furthermore, 10–12% of patients were staged incorrectly on planar and/or SPECT, and a substantial proportion of cases were equivocal on both planar and SPECT despite consensus reporting. SPECT/CT demonstrated a few additional advantages including the identification of uptake in benign Paget’s lesions (which were a source of false positive findings on planar and SPECT) and distinguishing nodal/soft tissue uptake from uptake in adjacent bone.

Conversely, in support of SPECT and planar imaging, although SPECT/CT did detect a greater number of patients with nodal, bone and visceral metastatic disease, the proportions of positive cases were not significantly higher. In addition, assessment of the primary tumour and seminal vesicle involvement is seldom the reason for referral for PS. In most patients PS is requested to rule out metastatic disease to guide management decisions. Thus the adequacy of SPECT alone and/or planar imaging has been demonstrated for PS. In addition, the adequacy of a limited field-of-view has been demonstrated. Consequently, there is potential to significantly reduce economic and material barriers to accessing PSMA radioligand imaging, especially in resource limited settings such as sub-Saharan Africa, where planar scintigraphy represents the majority of the diagnostic clinical nuclear medicine work done [20, 31, 32, 63, 64]. This will hopefully improve outcomes for patients with PCa in sub-Saharan Africa and other regions around the world that are equally impacted by resource limitation and outsized PCa mortality. Utilizing this resource-limitation-adapted PS imaging protocols, centres, across the developing world, may be able to leap-frog near the frontiers of modern PCa diagnostics with PSMA radioligand imaging (and potentially PSMA therapy and dosimetry), by “starting where they are, using what they have, and doing what they can” [65].

We acknowledge the limitations of our study. Foremost, the number of patients with metastatic disease in the cohort were small. Had the cohort been larger, or if the proportion with metastatic disease was higher, it is possible that clear superiority in the detection rates of SPECT/CT ± SPECT would have been demonstrated. Secondly, SPECT/CT was used as reference. Ideally, PS should be compared to PSMA PET/CT, however PSMA PET/CT was not routinely available during the study period. The additional advantages of SPECT/CT (e.g. in detecting benign osseus uptake and distinguishing bone from adjacent soft tissue uptake) were not quantified and their importance likely underestimated. The absence of histological confirmation of imaging findings is highlighted. Only a minority of the patients underwent radical prostatectomy after PS. This reflects clinical practice in our hospital, which is in part due to resource constraints, but also largely due to the advanced stage of the disease at presentation in many patients. Values for sensitivity and specificity of planar and SPECT could not be calculated due to the number of cases reported as equivocal for planar and SPECT. Ideally, the reviewers should have committed to reporting these cases as either positive or negative. Finally, the cohort comprised patients for staging of PCa as well as those being worked up for [^177^Lu]Lu-PSMA therapy, which ideally should be analyzed separately.

## Conclusion

The findings of this study confirm the known superiority of SPECT/CT over planar and SPECT imaging in PS. Hence, when PET/CT is unavailable, multi-FOV SPECT/CT is recommended. However, the lack of SPECT/CT capabilities should not preclude the use of PS in resource limited settings. Planar and SPECT imaging are both adequate and will correctly stage the majority of patients. Furthermore, time-based optimisations can be achieved by limiting the FOV to exclude the distal lower limbs. A salient future study would be a comparison of planar/SPECT PS to conventional imaging (e.g. prostate MRI, bone scan and CT scan) to see if PS without SPECT/CT confers significant benefit.

## Data Availability

The datasets used and/or analyzed during the current study are available from the corresponding author on reasonable request.

## Abbreviations

CT: Computed tomography
FOV: Field of View
HREC: Human Research Ethics Committee
PCa: Prostate Cancer
PET: Positron Emission Tomography
PET/CT: Positron Emission Tomography/Computed Tomography
PRLT: Peptide Receptor Radioligand Therapy
PS: [^99m^Tc]Tc-PSMA scintigraphy
PSMA: Prostate Specific Membrane Antigen
SPECT: Single Photon Emission Computed Tomography
SPECT/CT: Single Photon Emission Computed Tomography with Computed Tomography
USD: United States Dollars
